# Assessment of heat tolerance and identification of miRNAs during high-temperature response in grapevine

**DOI:** 10.3389/fpls.2024.1484892

**Published:** 2024-10-22

**Authors:** Lipeng Zhang, Yuanxu Teng, Yue Song, Junpeng Li, Zhen Zhang, Yuanyuan Xu, Dongying Fan, Lujia Wang, Yi Ren, Juan He, Shiren Song, Xiaojun Xi, Huaifeng Liu, Chao Ma

**Affiliations:** ^1^ Key Laboratory of Special Fruits and Vegetables Cultivation Physiology and Germplasm Resources Utilization of Xinjiang Production and Construction Corps, Department of Horticulture, Agricultural College of Shihezi University, Shihezi, China; ^2^ Shanghai Collaborative Innovation Center of Agri-Seeds, School of Agriculture and Biology, Shanghai Jiao Tong University, Shanghai, China; ^3^ College of Landscape and Horticulture, Yunnan Agricultural University, Kunming, China; ^4^ Forestry and Pomology Research Institute, Shanghai Academy of Agricultural Sciences, Shanghai, China

**Keywords:** heat stress, grapevine, next-generation sequencing, miRNA, Vvi-miR3633a

## Abstract

With global warming, heat stress has been recognized as a significant factor limiting grapevine development and fruit quality. MicroRNAs (miRNAs) are a class of small non-coding RNAs known to play crucial regulatory roles in stress resistance. Hence, there is an immediate requirement to cultivate and identify grapevine varieties that are resistant to heat and explore miRNA-mediated heat stress defense mechanisms. In this study, we assessed the thermal resistance of 38 grape germplasm resources and identified a series of miRNAs involved in heat stress resistance. The CK (25°C) and HS (45°C) groups of “Shenyue” cuttings of grapes were used as experimental materials for next-generation sequencing and construct libraries of small RNAs. A total of 177 known and 20 novel miRNAs were detected in the libraries. Differential expression analysis identified 65 differentially expressed miRNAs (DEMs) using the DE-Seq procedure. Furthermore, RT-qPCR validation confirmed complementary expression profiles of eight DEMs and their target genes between the HS and CK groups. Heterologous transformation further identified the function of Vvi-miR3633a downregulated under heat stress in *Arabidopsis*. In the heterologous expression lines, the survival rate was reduced by high temperature treatment indicating the ability of Vvi-miR3633a to regulate heat resistance. Assessing the heat resistance of grape species and the expression patterns of miRNA in response to high temperatures may reveal the molecular processes of heat resistance regulation mediated by miRNA in grapes under heat stress.

## Introduction

The *Vitis* species is widely planted worldwide and holds significant economic value (Gao et al., 2019; Chen et al., 2020; Zhang et al., 2021). Due to natural evolution and interspecific hybridization, different grape species exhibit varying levels of stress resistance (Zhang et al., 2023b). In recent years, global temperatures have been on the rise. According to the Intergovernmental Panel on Climate Change (IPCC) assessment report, the global temperature has increased by 0.85°C in the past 30 years (Hasanuzzaman et al., 2013). This increase in temperature has led to heat damage in many grape-producing areas, particularly during the summer when extremely high temperatures are experienced (Zha et al., 2021). Certain cultivation areas have witnessed temperatures at noon that exceed the threshold for grape survival leading to serious harm to grapevine development and fruit yield (Zha et al., 2016).

At present, there are several indicators utilized to assess plant thermal damage. These include changes in the external appearance of plants, chlorophyll fluorescence parameters, and various physiological and biochemical markers (Mittler et al., 2011). Chlorophyll fluorescence parameters are particularly important for assessing photosynthetic capacity under heat stress. For example, D. Stefanov et al. assessed the heat resistance of beans using chlorophyll fluorescence parameters, while grape species heat resistance based on the Fv/Fm value was identified (Stefanov et al., 2011).

As the non-coding RNA of 18–22 nt, miRNA has an irreplaceable role in targeting splice mRNA degradation or inhibition of post-transcriptional translation silencing mechanisms (Li H. et al., 2020; Ma et al., 2015, 2014). With the utilization of a sequencing platform for small RNA and enhancements in resequencing data, more and more miRNAs in horticultural plant genomes have been identified (Cheng et al., 2023; Bai et al., 2016; Chen et al., 2019; Saminathan et al., 2016). Initially, miRNAs were found in model plants such as *Arabidopsis thaliana*. Subsequently, as the study progressed, the miRNA libraries in plants encompassed both conserved and non-conserved miRNAs (Yu et al., 2017). In general, the expression levels of these non-conserved miRNAs were lower than those of conserved miRNAs.

As more plant genomes are sequenced, an expanding array of miRNAs are being identified and studied (Ding et al., 2020; Guan et al., 2013). miRNAs have been found to regulate the development pathways of multiple organs in plants (Knauer et al., 2013; Xu et al., 2007; Wang et al., 2019). Numerous researches have indicated the involvement and essential functions of miR156, miR164, and miR167 in the development of roots and leaves (Liu et al., 2022; Zhang et al., 2022; Ma et al., 2021). miR159, miR172, and miR319 mediate flowering time and petal size (Palatnik et al., 2007; Zhu and Helliwell, 2011). In the context of global warming, many researchers have identified plant miRNAs involved in high-temperature response mechanisms. In *Arabidopsis thaliana*, miR398 positively regulates heat tolerance by regulating target genes such as *CSD1* (*COPPER/ZINC SUPEROXIDE DISMUTASE 1*), *CSD2* and *CCS* (*COPPER CHAPERONE FOR SOD*) (Zhu et al., 2011). In the same way, the miR166-*PHB* (*PHABULOSA*) module increases the heat resistance of plants, as reported (Li et al., 2023). miR396 controls the expression of *HaWRKY6*, thus assisting sunflowers in defending against high-temperature stress (Giacomelli et al., 2012). Moreover, studies on some key coding RNAs impacting grapevine development under heat stress have been performed, such as miR160 and miR167 target multiple ARF (auxin response factor) genes to regulate thermal stability (Zhang et al., 2023b).

To date, databases of abiotic stress-related miRNAs have been established in a variety of horticultural plants based on high-throughput data (Sunkar and Zhu, 2004). In the corresponding species, conserved and heat stress-specific miRNAs have been identified. Although miRNA takes part in high-temperature responses in many species, the expression of miRNA varies among different species. Previous studies have identified the miRNA expression of “Thompson Seedless’ grape plantlets. However, the miRNA among different grape varieties in response to high temperature needs to be investigated further.

This study was based on heat resistance evaluation of germplasm resources and sRNA sequencing to explore the miRNA associated with high-temperature response. The results of the heat-treatment group (HS) and control group (CK) were analyzed. A total of 65 microRNAs were found to exhibit significant variances in expression levels. Annotation analysis of their potential target genes indicated that most of the genes may be related to molecular functional pathways. Compared with WT, high temperature decreased the tolerance of overexpressed *Arabidopsis*. This confirmed that Vvi-miR3633a can regulate the function of high-temperature response. In this study, the heat tolerance of 38 germplasm resources was identified. Then, the mechanism of the high-temperature response was analyzed based on physiological indexes and miRNA expression levels.

## Materials and methods

### Materials and treatments

Cuttings of 38 grapevine genotypes (wild species and cultivars) were grown in the greenhouse flowerpot of the College of Agriculture and Biology, Shanghai Jiao Tong University. The population origin and growth of these test materials are shown in **Table 1** and **Supplementary Figure S1**. There are 12 grape cuttings per genotype for a total of 456 plants. The grape plantlets were treated at 45°C for 0, 2, 4, and 8 h, and the mature leaves were collected. Each treatment was replicated within three distinct biological sets.

**Table 1:** Thermostability of 38 grape cultivars or species evaluated based on the Fv/Fm value.

Number	Cultivars	Species	Fv/Fm	Heat tolerance
1	Squire Seedless	*V. vinifera* L. × *V. labrusca*	0.632	
2	Selecta	*V. vinifera* L.	0.664	Cluster 4
3	Thompson Seedless	*V. vinifera* L.	0.677	
4	Hupei No. 1	*V. vinifera* L. × *V. labrusca*	0.705	
5	Seibel Blanc	*V. vinifera* L. × *V. labrusca*	0.710	
6	BK	*V. vinifera* L. × *V. labrusca*	0.716	
7	Jumeigui	*V. vinifera* L. × *V. labrusca*	0.719	
8	Sakashura Grape	*V. vinifera* L. × *V. labrusca*	0.723	
9	Zuijinxiang	*V. vinifera* L. × *V. labrusca*	0.723	Cluster 3
10	PvII2097	American wild grapes	0.727	
11	Heihuxiang	*V. vinifera* L. × *V. labrusca*	0.728	
12	Fushizhihui	*V. vinifera* L. × *V. labrusca*	0.730	
13	Concord Chatauqua	*V. vinifera* L. × *V. labrusca*	0.735	
14	America	*V. vinifera* L. × *V. labrusca*	0.741	
15	Hupei No. 2	*V. vinifera* L. × *V. labrusca*	0.742	
16	Salle Creek Grapes	American wild grapes	0.742	
17	Kaokebangdasi	*V. vinifera* L.	0.746	
18	Paulsen 1447	*V. vinifera* L. × *V. labrusca*	0.752	
19	Niagara Grape	American wild grapes	0.754	
20	Fujiminori	*V. vinifera* L. × *V. labrusca*	0.757	Cluster 2
21	Agate Grape	*V. vinifera* L.	0.759	
22	Kozma Pal Muskotaly	*V. vinifera* L. × *V. labrusca*	0.761	
23	Shenhua	*V. vinifera* L. × *V. labrusca*	0.763	
24	225 RU	American wild grapes	0.767	
25	White Lachaki	*V. vinifera* L.	0.769	
26	Zheng Guo No. 6	American wild grapes	0.775	
27	Shenfeng	*V. vinifera* L.	0.778	
28	Brillant	*V. vinifera* L. × *V. labrusca*	0.791	
29	Spentan Seedless	*V. vinifera* L. × *V. labrusca*	0.793	
30	Magaratch Ruby 56	*V. vinifera* L.	0.797	
31	Cuihong	American wild grapes	0.798	
32	Khalili Noir	*V. vinifera* L.	0.801	
33	Molixiang	*V. vinifera* L. × *V. labrusca*	0.801	Cluster 1
34	Shenyue	*V. vinifera* L. × *V. labrusca*	0.805	
35	Ziyun Niagara Grape	American wild grapes	0.809	
36	St. Pepin (Es282)	*V. vinifera* L. × *V. labrusca*	0.814	
37	Aromatic Rachaki	*V. vinifera* L.	0.814	
38	775(p)	American wild grapes	0.815	

The “Fv/Fm values” represent the average Fv/Fm values identified at 0, 2, 4, and 8 h under heat treatment.

*Arabidopsis* ecotype Col-0 was cultivated in a growth chamber of 22°C with a 16-h light and 45 μmol m^−2^ s^−1^ of photosynthetically active radiation (PAR), 8-h dark photoperiod. Seeds on a mixture of vermiculite and nutritive soil were sown. The 40-days olds of both wild-type (WT) and transgenic lines were exposed to a 24-h treatment at a temperature of 45°C. After the end of heat treatment, the thermal damage rate was calculated.

### Determination of physiological indicators

Fv/Fm values and fluorescence images were measured using Handy-PEA and Image-PAM (Device model). CAT, POD, and MDA contents were detected using the total Assay Kit (Sangon, China).

### Extraction of RNA and synthesis of cDNA

The enhanced CTAB technique was utilized for the extraction of total RNA (Li et al., 2009). Total DNA was removed by the DNase I (Invitrogen, CA, USA) kit. Based on the agar gel (1.0%) electrophoresis experiment, the practicability of RNA was verified. The concentration of RNA was detected by NanoDrop 1000 (Thermo, USA) instrument, which was subsequently used for small RNA transcriptome sequencing. For cDNA acquisition, 2 μg of RNA was subjected to reverse transcription using a Super Script RT (reverse transcriptase) kit from Invitrogen, CA, USA. miRNA levels were assessed using RT-qPCR, and cDNA was generated using Vazyme (China) miRNA RT enzyme Mix.

### Construction of small RNA library and analysis

The construction and analysis of sRNA sequencing libraries mainly include the following processes (Cao et al., 2018). Total RNA was extracted from the grape leaves at the CK (0 h, 45°C) and the HS (4 h, 45°C) for small RNA sequencing. Enrichment of low-molecular weight RNA was based on PAGE gel (15%). Splice sequences were introduced at the 5′ and 3′ termini of the RNA, after which specific splice primers were used for RT-PCR amplification. The sequencing analysis was conducted by Personalbio (Nanjing) Biotechnology Co., Ltd. on the Illumina PE 150 platform, with each sample undergoing three separate biological replicate sequencing analyses.

### Bioinformatic analysis of miRNAs

For the sequencing analysis of miRNA, the original data are first spliced removed and mass filtered, and the filtered sequences are de-processed (that is, the identical sequences are combined, and the corresponding abundance of the sequences is recorded). Then, the de-duplication sequence is compared with the Rfam database to obtain the annotation information of various sRNAs. In addition, regarding the reading of pre-miRNA/miRNA sequences, a two-nucleotide mismatch is allowed. Then, conserved miRNAs were obtained from the identified pre-miRNA/miRNA corresponding reads corresponding to the miRBase database (version 22.0). The predicted novel miRNA is obtained based on the search for complementary sequence star miRNA (miRNA*) in the precursor molecule in the sRNA library. Then, unfold software was further used to predict all the identified and potential secondary structure pre-miRNAs in the grape genome (Zuker, 2003). Ultimately, a newly discovered miRNA meeting the criterion of having a minimum folding energy index (MFEI) exceeding 0.85 is chosen (Bonnet et al., 2004).

### Analysis of differentially expressed miRNAs

All conserved miRNA expression data were collated, and the DE-Seq software (version 1.18.0) was utilized for analyzing the variance in miRNA expression levels. Diverse conserved miRNAs were identified based on their expression (|fold change| > 1) and the statistical significance of the expression variation (p-value < 0.05).

### miRNA target gene prediction and GO analyses

The target gene mRNA prediction of miRNA is mainly bound to the target site through complementary base pairing. The miRNA target in grape leaves was predicted based on the psRNA Target tool (http://plantgrn.noble.org/psRNATarget/). This includes reading all miRNAs into the FASTA file format. Next, known target genes were selected in the transcriptomic database of *Vitis vinifera*. According to the mRNA corresponding to differentially expressed miRNAs, functional analysis was performed using BLASTN (https://phytozome-next.jgi.doe.gov/). Subsequently, utilizing the gene ontology (GO) database available at http://www.geneontology.org/, assumptions were made regarding the molecular function, biological process, and cellular component. The genes mentioned above are associated with specific GO entries in the database through a calculation of their respective percentages. Any GO term exhibiting a p-value < 0.05 is deemed to be significantly enriched among the predicted target genes.

### Quantitative time quantitative PCR analysis

Based on the manufacturer’s instructions, the total RNA was converted to cDNA using oligo dT primers with FastKing RT Mix (Vazyme, China). RT-qPCR reactions were carried out in 10 μl using SYBR Green Super-mix (Vazyme) for analyzing mRNA and miRNA expression. The 2^−ΔΔCt^ method was employed for calculating gene expression levels (Livak and Schmittgen, 2001). All primer details are provided in **Supplementary Table S1**.

### Vector construction

The design of the VvimiR3633a overexpression vector was carried out using the pHb binary plasmid under CaMV35S promoter as a foundation. First, fragments of the pre-miRNA sequence were cloned into BamH І and Xba І linear vectors. Based on the Agrobacterium transformation method, the constructed plasmid was transformed into a GV3101 strain. At the same time, the antibiotics were cultured on LB medium with 50 mg·L^−1^ of kanamycin (Kan) and 25 mg·L^−1^ of rifampicin (Rif) and cultured at 28°C for 2–3 days. Next, the selected single clone was incubated in LB liquid medium of 25 mg·L^−1^ of Rif and 50 mg·L^−1^ of Kan in a shaker for 8–10 h, and the parameters were set at 200 rpm and 28°C.

### *Arabidopsis* transformation

*Arabidopsis* transformation according to the floral dipping method was used (Clough and Bent, 2008). Transgenic lines were screened by spraying T1 seedlings with 0.01% Glufosinate ammonium. Simultaneously, the positive transgenes and their expression levels were further verified based on PCR and RT-qPCR reactions. The primers of amplified sequences are referred to in **Supplementary Table S1**.

## Results

### Heat tolerance evaluation and germplasm classification

To assess the tolerance of different grape species to heat stress, 38 wild species and cultivars were placed in incubators. These plantlets were treated for 8 h at 45°C. At the same time, the Fv/Fm values of the leaves were measured at 0, 2, 4, and 8 h, respectively. Utilizing the ordered sample optimal segmentation clustering technique, we categorized the mean Fv/Fm values of chlorophyll fluorescence parameters, thereby deriving the optimal segmentation error function and heat resistance classification outcomes for 38 grape germplasm (**Table 1**). Subsequently, a scatter coordinate diagram was constructed using the partitioned error function and the cluster number. As depicted in the broken line graph (**Supplementary Figure S2**), the inflection point was identified to be between 4 and 5. With the increment of the cluster number, the error function exhibited a trend toward stability. At the same time, in view of the small span range of the Fv/Fm of grape heat resistance, it was initially classified into four grades. This classification was further refined by delineating four distinct intervals of Fv/Fm thresholds, viz., (0.63, 0.67), (0.70, 0.74), (0.74, 0.78), and (0.79, 0.82) (**Supplementary Table S2**). Based on this criterion, 11 grapevine cultivars with heat stress resistance and three sensitive cultivars were identified. The germplasm resources with high heat resistance included “Shenyue” (*V. vinifera* × *V. labrusca.* L), “Cuihong” (America wild grape), and “775 (p)” (America wild grape) (**Table 1**). Those results may serve as a theoretical foundation for the development of heat-resistant grape varieties.

### Phenotypic and physiological responses of “Shenyue” to heat stress

**Figure 1:**
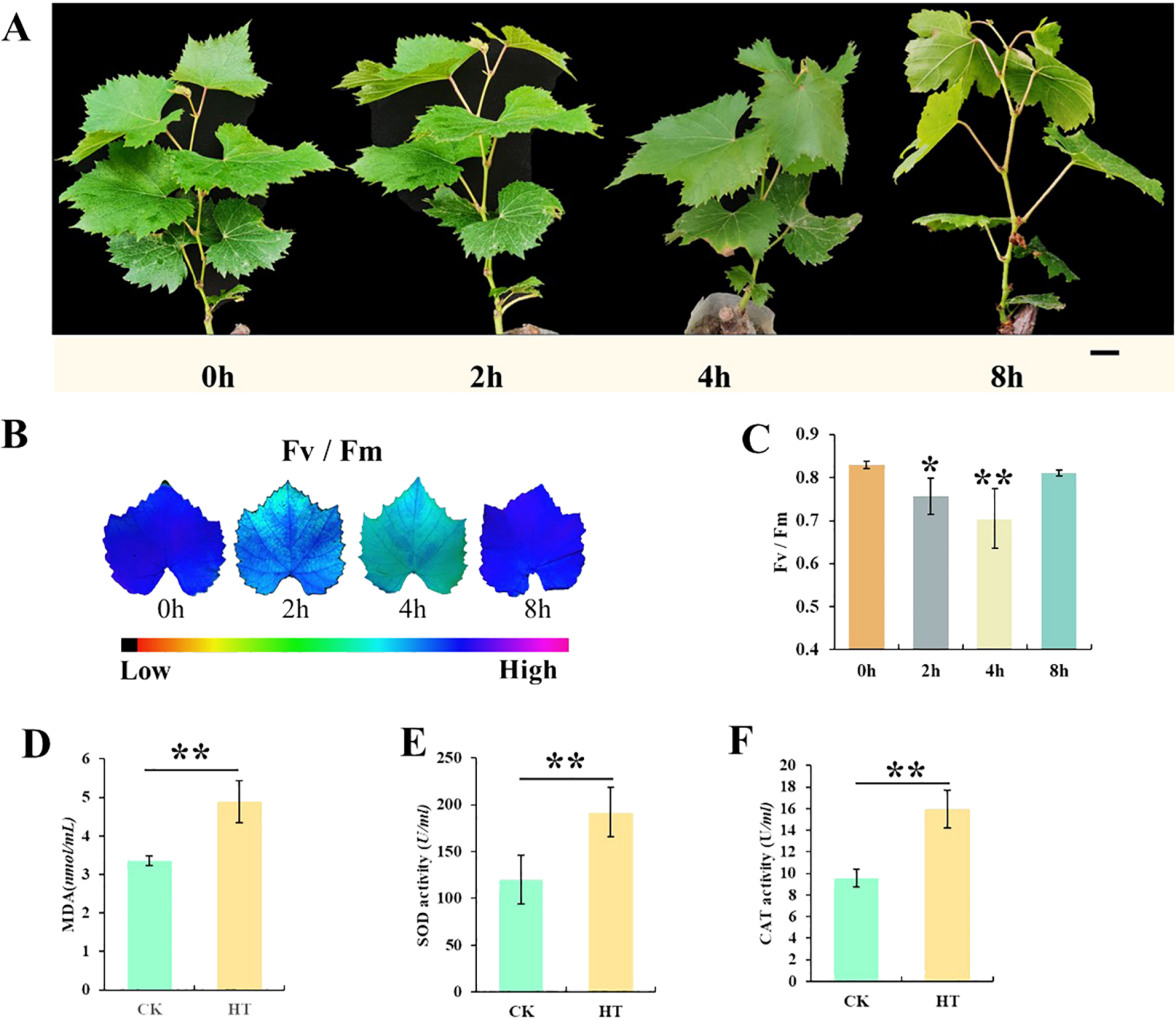
Character changes of “Shenyue” variety under heat stress. **(A)** Phenotypes of “Shenyue” variety subjected to heat tolerance. **(B)** Fluorescence imaging and **(C)** Fv/Fm values. **(D–F)** MDA (malondialdehyde) contents, SOD (superoxide dismutase) and CAT (catalase) activity in the leaves treatment group and control group. Bar = 1 cm. *, **: By two-sided test, P < 0.05, 0.01 were significant.

To delve deeper into the mechanism of thermostability in grapes in response to heat stress, we analyzed the efficiency of PS II and the changes in REDOX substances. During heat stress treatment, after HS for 2 and 4 h, there was no significant change in the “Shenyue” grape leaves. However, when the duration reached 8 h, the plants began to wilt, which indicated that growth and development were inhibited (**Supplementary Figure 1A**). Although the Fv/Fm value decreased after 2 and 4 h, it was increased from 4 to 8 h and reached the normal value (0.82, no significant difference compared with 0 h). These findings indicated that the plants were able to adapt to heat resistance and develop normally after 8 h (**Figures 1B, C**). Notably, besides the Fv/Fm value, CAT enzyme activity, POD activity, and MDA content were also indicators to evaluate the ability of plants to cope with abiotic stress (Zhang et al., 2023a). During heat stress treatment, CAT, POD activity, and MDA content in HS (4 h, space at 45°C) showed an increasing trend compared with CK (0 h) (**Figures 1D–F**). The increase in oxidase activity indicates that heat stress activates a regulatory mechanism inherent in heat-resistant varieties accelerating the synthesis of oxidase to achieve rapid removal of harmful substances (**Figure 1**).

### Small RNA sequencing and annotation

The quality determination found that heat treatment for 4 h was the key point for phenotype and physiological characteristics. To explore the impact of heat tolerance on grapevine growth, we created small RNA libraries from CK and HS leaf samples. Cluster analysis and principal component analysis (PCA) of sRNA expression revealed consistent results across the three biological replicates in both libraries (**Supplementary Figures S3C, D**). By deep analysis, 15,335,614 and 16,209,648 unfiltered reads were obtained from CK and HS, respectively (**Supplementary Table S3**). After excluding low-quality sequences, 5′ and 3′ adapters, and screening conditions shorter than 18 nucleotides from two libraries, a total of 11,020,874 (CK) and 13,663,198 (HS) unique sequences could be mapped to the grape genome (**Supplementary Table S3**). The distribution of sRNA length is similar among CK-1, CK-2, HS-1, and HS-2, where 21 nt sRNA is the main peak, followed by 24- and 22-nt classes (**Supplementary Figure S3B**). Next, the repetitive sequences were filtered, and a Venn diagram showed 2,725,443 (CK) and 1,804,971 (HS) unique sequences and 652,793 common sequences (**Supplementary Figure S3A**).

Using the Blast program, unique reads were compared with the Rfam13 database to screen four known classes of ncRNAs, including rRNA, tRNA, snRNA, and snoRNA (**Supplementary Figure S4**). The screening criteria are no more than two perfect matches or mismatches. Comparison results showed that CK-1 and HS-1 had 63.4% and 63.1% matching sequences of rRNA, tRNA, snRNA, Cis-reg, and other non-coding RNA, respectively (**Supplementary Figure S4A**; **Supplementary Table S4**). The identified miRNAs were annotated using miRBase (version 22.0), and only 0.26% and 0.19% of the distinct sequences were recognized as miRNAs in HS and CK, respectively (**Supplementary Figure S4B**; **Supplementary Table S5**). The distribution of classified total sRNA and unique sRNA is illustrated in **Supplementary Figure S4**. HS-1 and CK-2 had similar overall distribution patterns, with rRNA being the majority of the total sRNAs, while the number of miRNAs in the CK-1 group was the largest.

The base preference of miRNA may be different at different locations. Statistical analysis was performed for base composition bias at the first position of the 5′ end and base bias at each position, and it was found that 19– to 24-nt miRNAs start with 5′ U (Uracil), particularly 20-nt sRNAs (**Supplementary Figure S5**). This is consistent with the research reports (Li H. et al., 2020), which is also one of the classical features of miRNAs. Furthermore, when examining nucleotide bias in 25-nt miRNAs at each position, it was observed that nucleotide U appeared most frequently at position 1, with position 25 following closely behind.

### Known and novel miRNA identification

Unannotated reads were compared with mature miRNA sequences from all plant species in miRbase v 22.0 using Blast to identify conserved miRNAs. The screening criteria required a perfect match or a mismatch of no more than two nucleotides. A total of 477 known miRNAs from 69 families were detected in grape leaves (**Supplementary Table S6**). These miRNA families are also conserved in a variety of plants, such as miR166, miR396, and miR396 (Sunkar and Zhu, 2004). In the existing small RNA collection, Vvi-miR169 had the highest count of miRNAs with 23 members, while Vvi-miR395 followed closely with 13 members. Families represented by a single member include Vvi-miR447 and Vvi-miR528 (**Supplementary Table S6**).

To discover novel miRNAs, we compared the sequence to the Pinot Noir genome (PN40024), but did not annotate any information. Then, the Mireap program was used to predict novel pre-miRNAs, and RNAfold was used to draw secondary structure maps. The examination indicated that the sRNA sequences could form stem-loop structures, as demonstrated in the selected portion (**Supplementary Figure S6**). During the exploration for matching miRNA* sequences, all 421 potential miRNAs were classified as newly discovered miRNAs (**Supplementary Table S7**).

To compare miRNA expression patterns in the two libraries, the read of each miRNA was standardized to TPM (transcripts per million). We found significant differences in the expression abundance of each miRNA, ranging from 6,818.56 to 0 TPM, with Vvi-miR3634-3p, aof-miR166d, and ppe-miR482b-5p being the most representative miRNA families (**Supplementary Table S8**). For novel-miRNAs, the abundance of vvi-m1820-3p was the highest in the HT-1 library followed by Vvi-m1784-3p and Vvi-m2634-5p. In addition, these three novel-miRNAs also appeared widely in CK libraries, but the difference was that the abundance of Vvi-m1820-3p ranked lower than that of Vvi-m1784-3p (**Supplementary Table S7**).

### Different expressions of miRNAs in CK and HT

**Figure 2:**
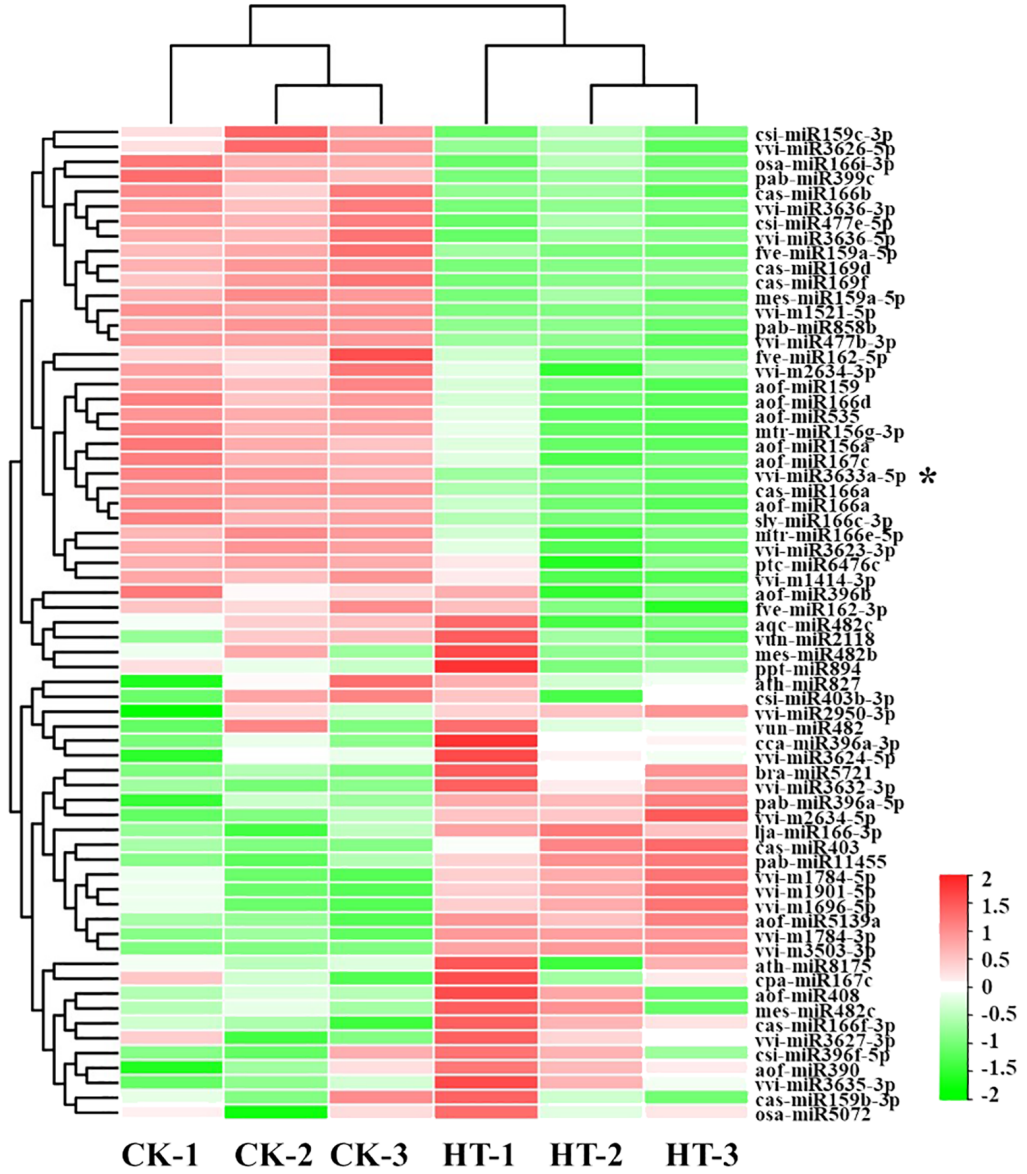
Hierarchical clustering of differently expressed miRNAs in leaves under heat stress. Horizontal represents miRNA genes and vertical columns represent individual samples. Red and green represent miRNAs with high and low expressions, respectively. * Represents: Vvi-miR3633a with heterologous expression function verification.

The occurrence times can be used as an indicator to evaluate the relative expression abundance of miRNA. Based on this, we collated the conserved miRNA expression data of each sample and used the DESeq software (version 1.18.0) to analyze the differentially expressed miRNAs in the two libraries. For comparison of CK and HS sRNA libraries, 65 miRNAs were differentially expressed (|fold change| > 1, p < 0.05), among which 32 miRNAs were upregulated, and 33 miRNAs were downregulated (**Figure 2**; **Supplementary Figure S6**). These results suggest that heat stress may affect the growth and development of grape by affecting miRNA expression patterns.

### Differential miRNAs were identified by RT-qPCR

**Figure 3:**
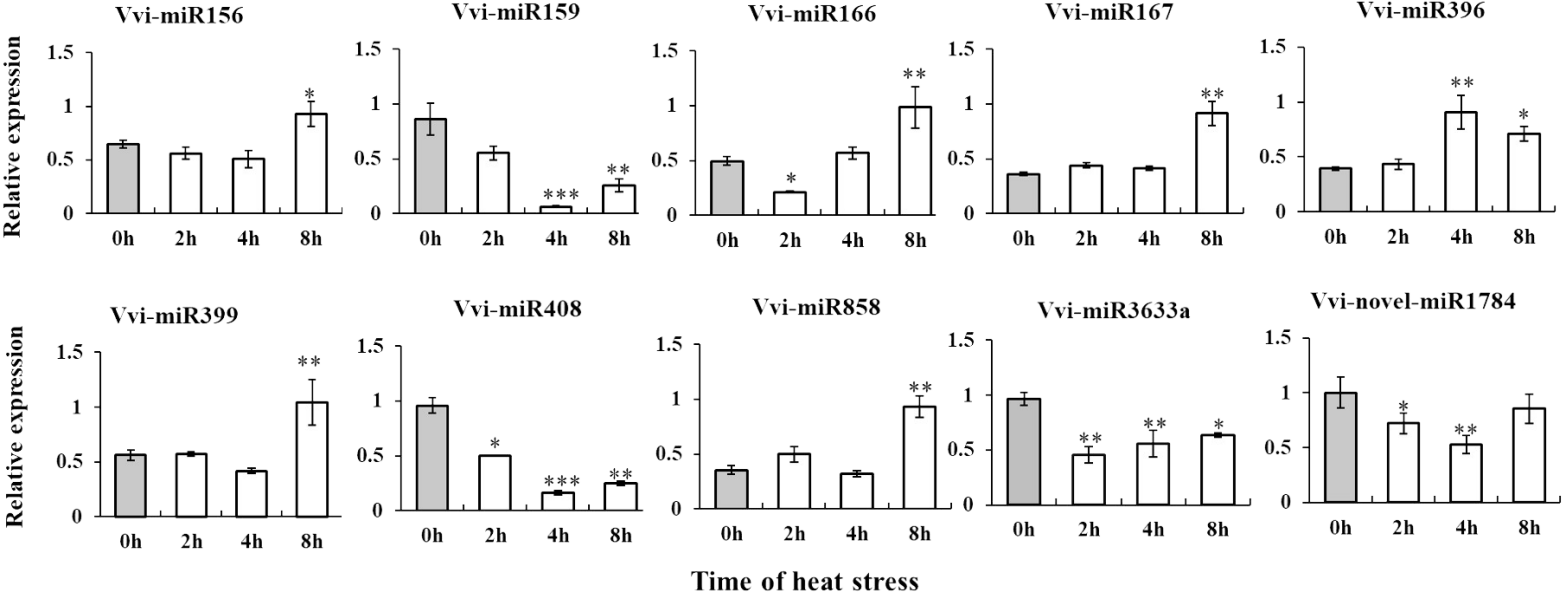
Identification and expression level analysis of differential miRNAs by stem loop RT-qPCR. *, **, ***By two-sided test, p < 0.05, 0.01, 0.001 were significant. The standard error between the three biological replicates is represented by the error line. The X-axis represents the time of heat stress, and the Y-axis represents the relative expression of Vvi-miRNA.

To explore the potential role of miRNA in grape responses to high temperatures and validate the variations in miRNA expression, we selected seven known miRNAs and one novel miRNA. Through RT-qPCR analysis, we observed that the expression patterns of these eight miRNAs in control conditions (CK) and under high temperature (HT) were consistent with the sequencing data. Additionally, we noted changes in miRNA expression levels over the course of heat stress (**Figure 3**). These results once again confirmed that the above differentially expressed miRNAs participate in the heat stress response and regulate the development of grapevines.

**Figure 4:**
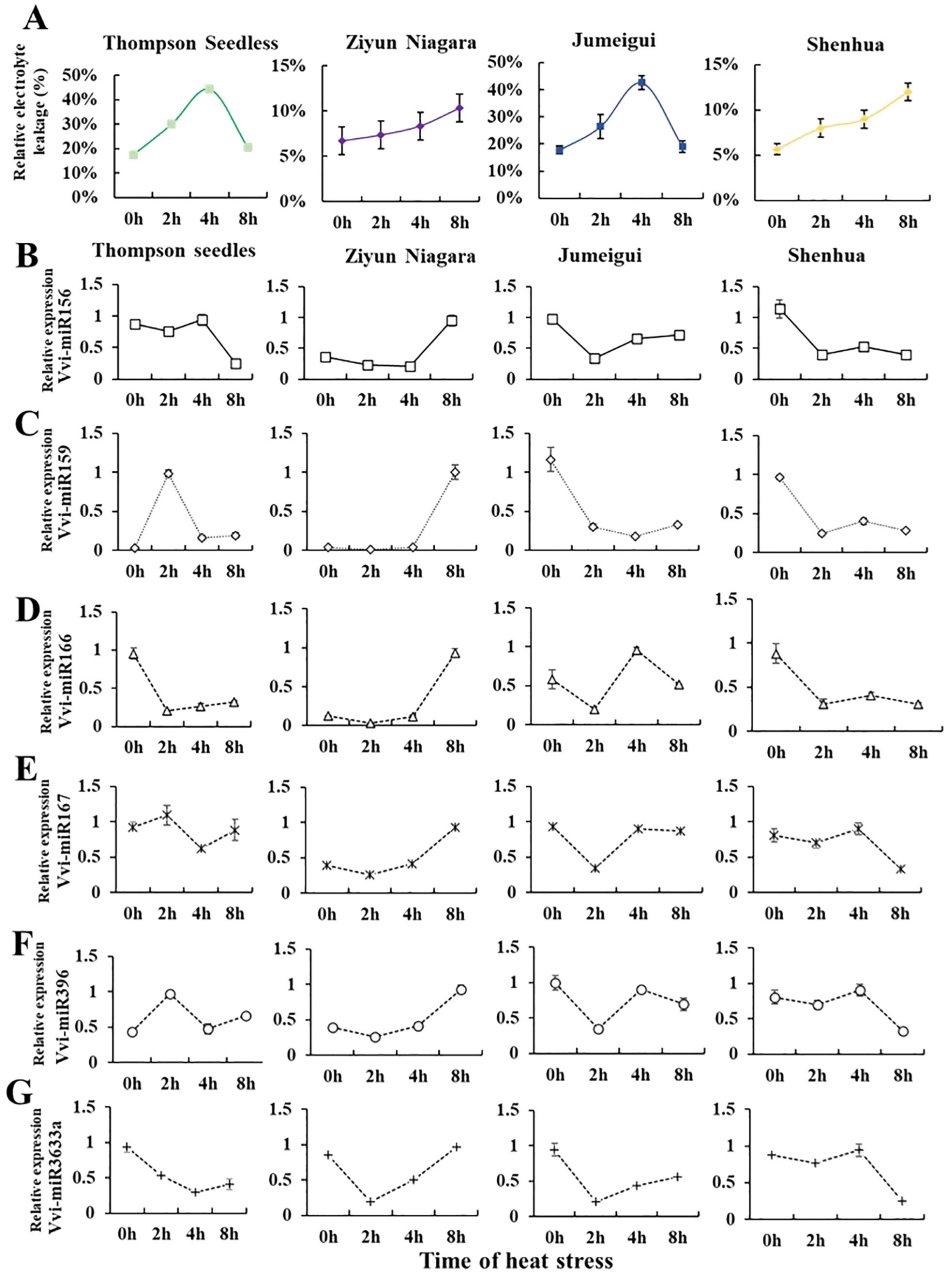
Analysis of relative electrical conductivity and miRNA expression in four grape varieties. **(A)** Indicates relative conductivity content in varieties such as “Thompson Seedless,” “Jumeigui,” “Shenhua,” and “Ziyun Niagara.” **(B–G)** The expression levels of VvimiR156, miR159, miR166, miR167, miR396, and miR3633a were identified in “Thompson Seedless,” “Jumeigui,” “Shenhua,” and “Ziyun Niagara.” The X-axis represents the time of heat stress, and the Y-axis represents the relative expression of Vvi-miRNA. *, **, ***By two-sided test, p < 0.05, 0.01, 0.001 were significant. The standard error between the three biological replicates is represented by the error line.

In the process of long-term evolution, *Vitis* plants have formed ecological habits to adapt to the natural environment, and the heat resistance of different species is quite different. To investigate whether the expression patterns of miRNAs were different in grapes of different heat tolerance clusters, based on heat resistance classification, the grape varieties “Thompson Seedless” (Cluster 4), “Jumeigui” (Cluster 3), “Shenhua” (Cluster 2), and “Ziyun Niagara” (Cluster 1) were selected for miRNA expression level detection. To assess the impact of heat stress on grapevine growth inhibition, we initially measured the relative electrical conductivity of these four varieties. The results indicated that the electrolyte content in the cell solution was increased under heat stress, and the conductivity of the cell solution was increased. However, the response varied over time among the varieties. The heat-resistant varieties “Ziyun Niagara” showed an increasing trend, while the sensitive varieties “Thompson Seedless” showed an increasing trend first and then a decreasing trend. Then, based on the results, Vvi-miR156, Vvi-miR159, Vvi-miR166, Vvi-miR167, Vvi-miR396, and Vvi-miR3633a, with significant differences under heat stress, were selected. RT-qPCR results indicated varied expression patterns with heat stress treatment duration (**Figure 4**). Notably, Vvi-miR3633a consistently displayed a downward trend across all varieties during heat stress treatment aligning with previous findings that Vvi-miR3633a negatively regulates the high-temperature response mechanism of grapes (Zhang et al., 2023a). Those results suggest that miR3633a is the core miRNA in the heat stress response mechanism.

### Prediction and annotation of targets of differentially expressed miRNAs

In plants, miRNAs bind to target mRNA by complementary base pairing, thereby inhibiting the expression of targeted genes (Ma et al., 2021). High-throughput sequencing results identified 65 differentially expressed miRNAs in HT and CK libraries, which may regulate different targets in grapevine response thermostability mechanisms. At the same time, most of the miRNA target genes are conserved in plants (Adai et al., 2005), including grapes. For example, in the above results, miR156 targets *SBP*/SPLs (squamosa promoter binding protein-like), miR166 targets *ATHB-15* and *HOX32* (homeobox-leucine zipper protein), miR169 targets NF-YA (nuclear transcription factor Y subunit A), miR396 targets GRFs (*growth-regulating factor 1/4/8*), and some auxin response factors ARFs (targets of miR167 and miR393) (**Supplementary Table S9**). Most of the functions of these genes encoding proteins are to regulate plant development and abiotic stress-related signal transduction.

**Figure 5:**
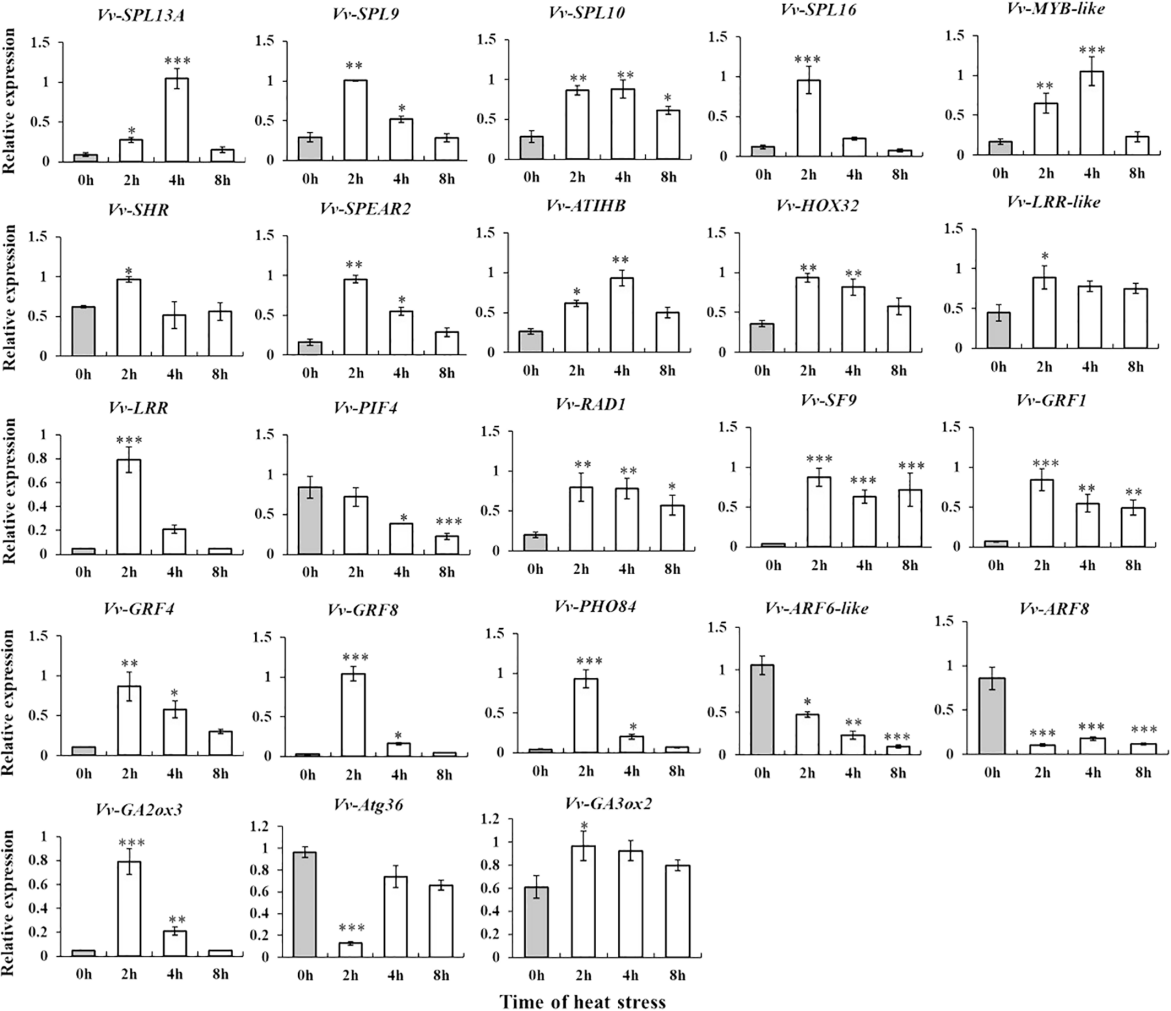
RT-qPCR examination was conducted to analyze the target genes of specifically expressed miRNAs. *, **, ***Statistical significance was observed at p < 0.05, 0.01, and 0.001 by t-test. The error bars represent the standard deviation of three biological replicates. The X-axis represents the time of heat stress, and the Y-axis represents the relative expression of target genes.

To better understand how the novel miRNAs function in grapes, we selected differentially expressed novel miRNAs (Vvi-m1414-3p, Vvi-m2634-5p, Vvi-m1901-5p, Vvi-m1696-5p, and Vvi-m1784-5p) for target gene prediction (**Supplementary Table S10**). The findings indicate that the majority of the recently discovered miRNA targets are associated with transcription factors specific (such as *SUPL13* and *RAM1-like*) to plants followed by metabolically related regulators (such as protein kinases, α/β hydrolases, and sucrose synthetase). In addition, there are several other targeted genes for immune- or stress-related functions. Then, the expression patterns of some differentially expressed miRNA target genes at four time points under high-temperature stress were identified by RT-qPCR. The results found that the expression trend of most target genes under heat treatment was complementary to the expression profile of miRNAs. Meanwhile, the expression level of some target genes showed the same trend with the continuous change in high-temperature time (**Figure 5**).

### GO and KEGG pathway analysis

**Figure 6:**
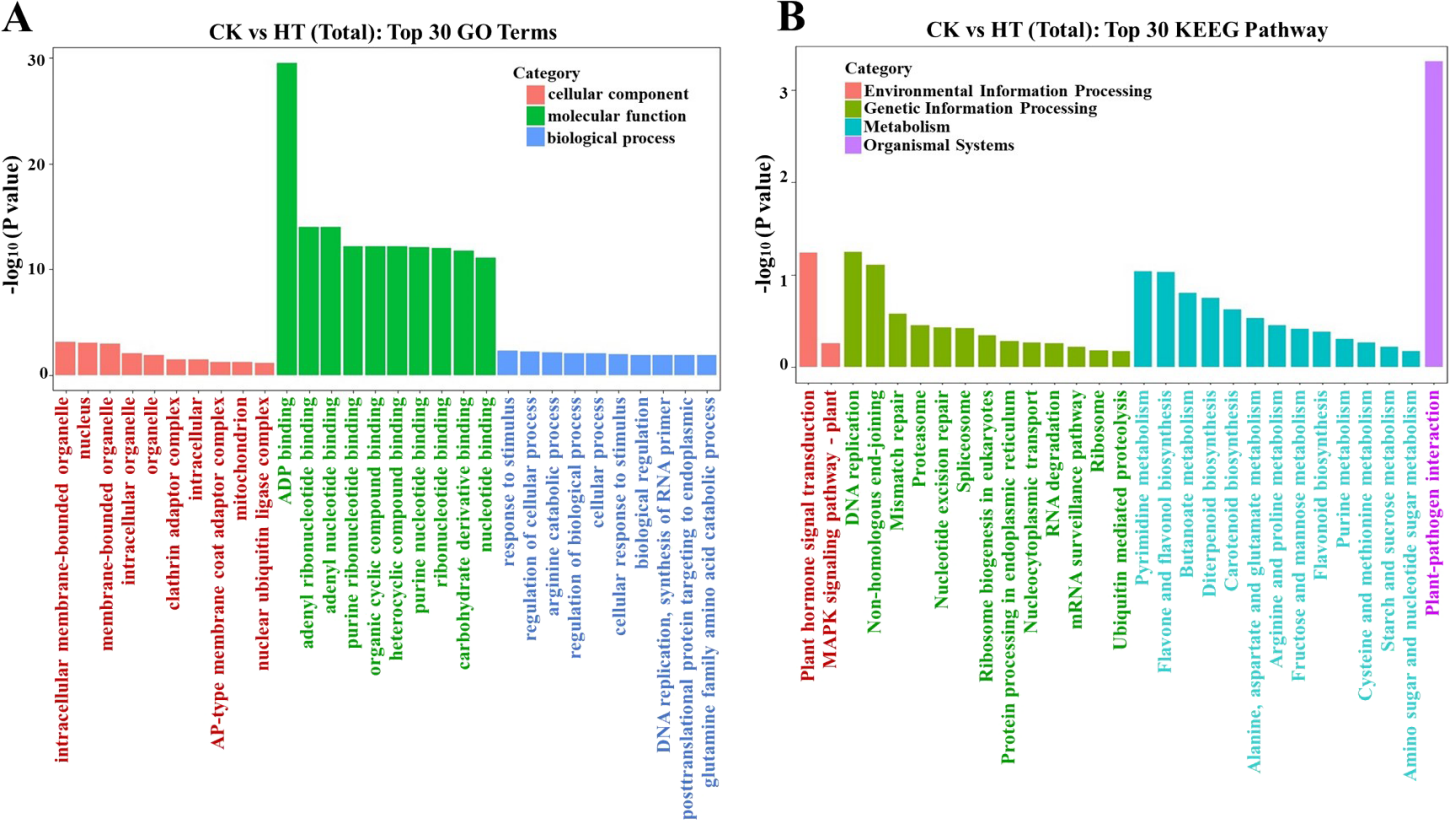
**(A)** Gene ontology (GO) and **(B)** KEEG pathway classification of target genes predicted by differential expression of miRNA (Top 30).

To elucidate the biological roles of miRNA target genes, the GO enrichment analysis findings for differentially expressed miRNA target genes were categorized based on molecular function (MF), biological process (BP), and cellular component (CC). The top 30 GO terms with the most significant enrichment (lowest p-value) were selected for each category. Among the cellular components, “intracellular” was predominant, followed by “intracellular organelle” and “membrane-bounded organelle.” Regarding molecular functions, ADP synthesis pathways were most abundant, followed by “organic cyclic compound binding” and “heterocyclic compound binding.” In biological processes, categories such as “cellular process” and “response to stimulus” were prominently enriched. Our GO analysis suggests that miRNAs target genes involved in plant development and stress responses, particularly aiding adaptation to heat stress (**Figure 6A**).

To investigate the integrity of miRNA transcriptomes, the hypergeometric distribution was employed to assess the significant enrichment pathway of target genes in comparison to the background of the Pinot Noir genome (PN40024). The KEGG enrichment analysis results of differentially expressed miRNA target genes were selected to reveal the top 30 pathways (that is, p-value < 0.05), the most significant enrichment (**Figure 6B**; **Supplementary Table S11**). These pathways include plant hormone signal transduction, MAPK signaling pathway, DNA replication, non-homologous end-joining, pyrimidine metabolism, fructose and mannose metabolism, and other major metabolic pathways; according to these candidate genes, the main enrichment is miR156, miR166, miR167, miR396, miR3633a, and Vvi-m1784-5p multiple miRNA family (**Supplementary Table S11**). Glucose-related pathways and biosynthesis of secondary metabolites also play an important role in grape high-temperature response. Additionally, novel miRNAs were found to be involved in different steps of multiple pathways, such as DNA replication and arginine and proline metabolism. At the same time, it is possible that some key hormone-related pathways are also involved in the synthesis of abiotic stress-related REDOX compounds.

### Phenotypic characterization of Vvi-miR3633a-OE lines in *Arabidopsis* under heat stress

**Figure 7:**
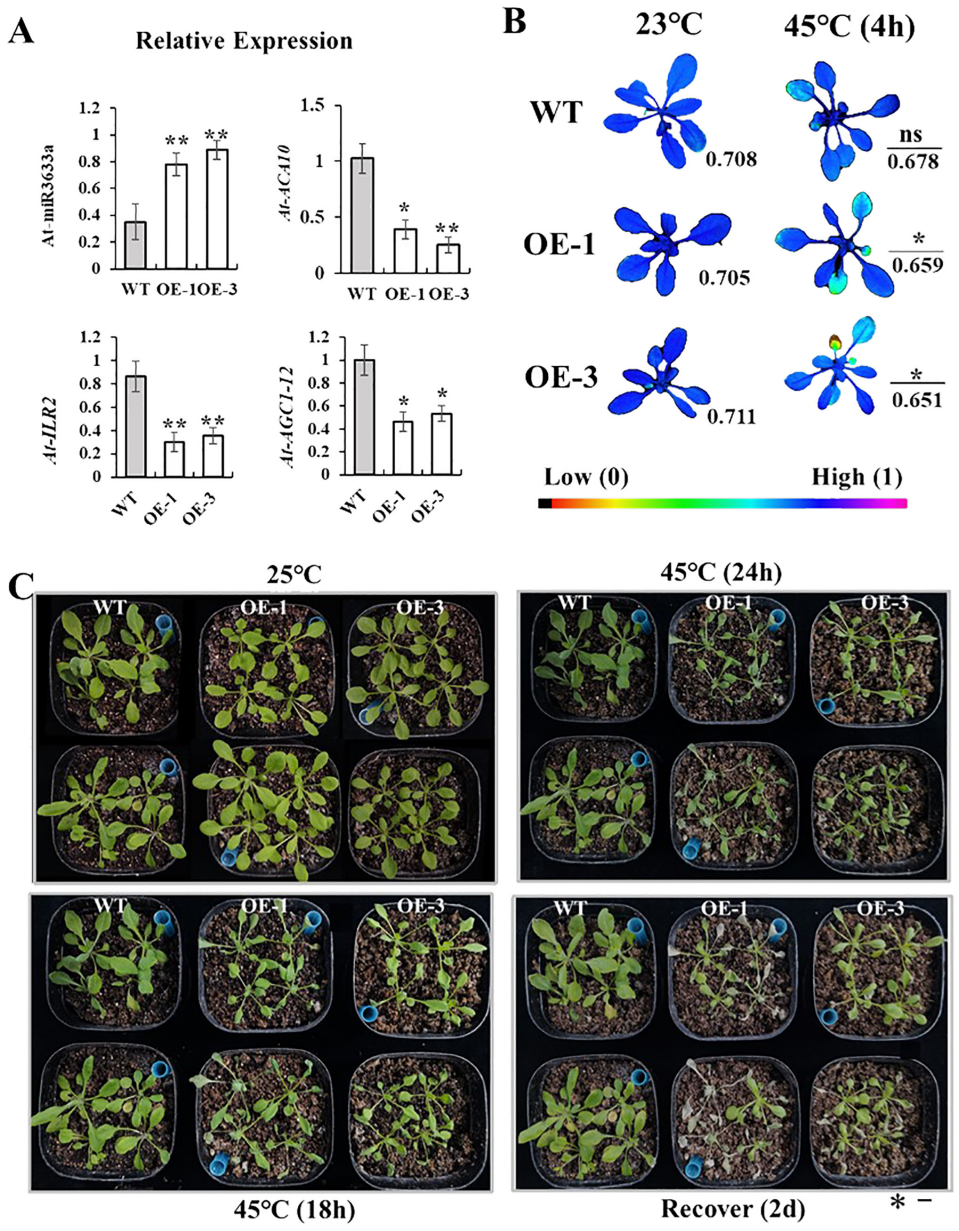
Phenotype characterization of transgenic *Arabidopsis*. **(A)** Relative expression of ath-miR3633a detected and target genes (*AtACA10*, *AtILR2*, and *ACG1-12*) by RT-qPCR. **(B)** Fv/Fm fluorescence photos changed at 45°C (4 h) and 23°C as control. **(C)** Phenotypic identification of WT and OE-miR3633a seedlings under heat stress and recovered for 2 days. The error bars represent the standard deviation of the three biological replicates. All data were subjected to t-test analysis (*p < 0.05; **p < 0.01; ns, no significant). The bar corresponds to a length of 1 cm.

Heat stress can inhibit the growth and development of grapevines. To decipher the function of differentially expressed miRNAs in plants, we conducted heterologous gene expression experiments. In addition, it has been reported that Vvi-miR3633a regulates peroxidase activity (Bai et al., 2022), and oxidase activity is an important indicator of plant heat damage. Based on *Arabidopsis* floral dip method, we overexpressed the Vv-pri-miR3633a sequence into Arabidopsis. The expression levels of ath-miR3633a and its target genes *AtACA10*, *ACG1-12*, and *AtILR2* in Vvi-miR3633a-OE and WT were determined by RT-qPCR. The results showed that the expression level of miR3633a in WT strain was significantly lower than that of Vvi-miR3633a-OE (**Figure 7A**). Its targets *AtACA10*, *ACG1-12*, and *AtILR2* were lower in overexpressed strains than in WT (**Figure 7B**). These results suggest that miR3633a may be involved in plant thermal response by regulating the expression of target genes. To further verify the assumption, the 4-week-old seedlings of the homozygous line were treated at 45°C for 24 h and then at 22°C for 2 days to recover. The results showed that compared with WT (**Figure 7C**), the heat resistance of OE-1/3 transgenic strain was weakened, and the Fv/Fm (maximum photosynthetic efficiency of photosystem II) was decreased (**Figure 7B**). Phenotypic observation of *Arabidopsis* after recovery confirmed the heat resistance results, with survival rates of OE1 and OE3 significantly lower than those of WT (**Figure 7C**, **Supplementary Figure S8**). It further indicated that the expression of Vvi-miR3633a negatively regulated the high heat resistance of plants.

## Discussion

Grapes, as an economic fruit tree (Gao et al., 2019, 2018), has an optimal growth temperature of approximately 30°C (Ye et al., 2020; Zha et al., 2016). As one of abiotic stresses, heat stress can directly or indirectly reduce grape fruit quality and grapevine development (Zha et al., 2020). Therefore, it is necessary to identify the heat resistance grade of grapes. The previous study found that 45°C heat stress could seriously damage the “Thompson Seedless” grape plantlets (Zhang et al., 2023). Based on this, we chose 45°C for heat resistance treatment in this study. The physiological and biochemical indexes of plants were affected by heat stress in different degrees (**Figure 1**). In addition, previous studies have identified methods for evaluating the heat damage of multiple varieties. For example, the chlorophyll fluorescence dynamic parameter method, which is simple, fast, and sensitive, can directly evaluate the degree of grape heat damage (Jiang et al., 2017). Herein, based on the determination of Fv/Fm values of 38 grape varieties under heat stress, the heat resistance was evaluated (**Table 1**). Meanwhile, the activity of SOD/CAT enzyme, MDA content, and relative conductivity of heat-resistant varieties were measured (**Figures 1D–F**, **4A**). These measures are well correlated with stress degree and can be used effectively for heat resistance evaluation.

Due to the diversity of genetic origins, we determined that grape varieties differ in their response to heat stress. “775 (p),” “Ziyun Aiagara” grapes, “Shenyue,” and “Cuihong” varieties are highly resistant to heat. However, “Thompson Seedless,” “BK,” “Jumeigui,” and “Zuijinxiang” are sensitive to high-temperature stress (**Table 1**). In addition, we found that wild species ranked high in heat resistance overall, higher than table grapes. The four heat resistance grades based on cluster analysis showed that the heat resistance of the varieties was not completely related to the origin of the varieties. For example, the American wild grapes were not completely higher than the *V. vinifera* × *V. labrusca* (**Table 1**). This is similar to previous research results to some extent (Zhang et al., 2023a). Compared with the previous research results, it not only perfected the heat-resistant variety resources but also enriched the high-temperature resistance detection methods. Of course, if we want to obtain more accurate heat resistance results for the species, we also need to conduct further research on the heat resistance of grape fruits, roots, stems, and flowers (Chen et al., 2010; Clepet et al., 2021). In summary, our study on the difference of physiological and biochemical indexes of grapes under heat stress provided a valuable basis for understanding the heat resistance of grapes.

MiRNA is a key center for regulating gene network pathways (Ding et al., 2020). It not only participates in plant growth and development but also plays an important role in the abiotic stress response (Chen et al., 2022; Ma et al., 2015). Although researchers have identified a range of grape-related miRNAs through second-generation sequencing, the mechanisms of miRNA response to high temperatures in grape leaves have been less reported. Herein, we identified the small RNA transcriptome of grape leaves under heat treatment and control. A 12× deep sequencing based on the next-generation sequencing (NGS) Illumina platform revealed a large number of conserved miRNAs and novel miRNAs. The differentially expressed miRNAs were also analyzed between heat stress treatment and control groups based on the DEG-seq software. Further analysis of the sRNA transcriptome of grape leaves showed that the length distribution of sRNAs was similar to that of published horticultural plants, such as apples (Ma et al., 2014) and citrus (Cheng et al., 2023), with 21- to 24-nt sRNAs accounting for the majority. Then, we found that 21-nt sRNAs accounted for the largest proportion, followed by 24-nt sRNAs (**Supplementary Figure S3**), which is consistent with the previous analysis of grapes (Zhang et al., 2023a). These results once again confirm the similarity of small RNA transcriptomes in plant species analysis.

Based on two sRNA libraries, 65 differentially expressed miRNAs were identified (**Supplementary Figure S7**). However, it is worth noting that the same miRNA family members were upregulated and downregulated simultaneously in different libraries, such as miR166 and miR159. We speculate that that there were differences in the promoter sequence of miRNAs, which affected the differences in miRNA expression (**Figure 2**). RT-qPCR detected some high-abundance differentially expressed miRNAs (**Figure 3**) further verifying the accuracy of high-throughput sequencing results. At the same time, some miRNAs were selected for further detection in four varieties of grapes, and the results showed that the expression trend of miRNAs in response to heat stress was different in different varieties, such as miR159 and miR3633a (**Figure 4**). It is speculated that this may be caused by the difference in motifs on the promoter and the difference in secondary structure sequence. The difference of these miRNAs in response to heat stress in different varieties can be developed as molecular markers for heat resistance identification and applied to molecular breeding of plants.

In horticultural plants, miRNA plays a regulatory role mainly by mediating mRNA cleavage (He et al., 2022). Therefore, to elucidate the role of miRNA in the grape high-temperature response, sequence prediction of target genes is necessary. In this research, we utilized the psRNA Target website to predict all target genes of miRNAs that were expressed differentially. Then, GO classification (**Supplementary Figure 6A**) and KEGG analysis (**Figure 6B**) were performed based on target genes and databases. These results indicated that the target genes were mainly involved in the ADP binding process and the plant–pathogen interaction pathway. The reason for the high frequency of ADP binding can be understood as that heat stress accelerates the electron transfer and ATP synthesis in plants. Although further validation is needed in grapes to elucidate the true targets, the annotation of these predicted miRNA targets could provide another perspective on heat stress-related gene regulation.

Due to the swift progress in molecular bioinformatics, numerous researches have indicated that miRNA plays a role in plants’ response to high temperature and their post-transcriptional development (Stief et al., 2014; Zhu et al., 2011). The expression of miR166 (Li et al., 2023), miR167 (Zhang et al., 2023a), and miR398 (Zhu et al., 2011) in plants can improve heat resistance. When miR398 (Li Y. et al., 2020) expression is absent, the heat tolerance of the plant is reduced. As a non-conserved miRNA family, the abiotic stress-related functional characteristics of Vvi-miR3633a have not yet been determined. In a previous study, we performed high-throughput sequencing on the quality of heat-treated Thompson Seedless tissue culture plantlets and Muscat fruits to determine the exact sequence of Vvi-miR3633a. Notably, the miR3633a sequence in the “Thompson Seedless” and “Shenyue varieties” was consistent with the sequence reported by miRbase. In addition, despite the consensus that Vvi-miR3633a is a specific miRNA of *Vitis* species, a researcher identified the expression of miR3633 during the development of camellia (Yin et al., 2016). These results suggest that Vvi-miR3633a may play a role in a variety of plants. Therefore, based on the Vvi-miR3633a sequence, we predicted its potential target genes, *AtACA10*, *AtILR2*, and *AtAGC1-12*, which are related to auxin synthesis (Kumar et al., 2020) and Ca^2+^ regulatory pathways (George et al., 2008). Most of these pathways are closely related to plant growth and development and abiotic stress. In the study, the comparison between CK and HS libraries found that there was a difference in the abundance of Vvi-miR3633a, and the RT-qPCR results confirmed that the expression level of Vvi-miR3633a under heat treatment was lower than that in the control group (**Figures 3**, **4**). The heterogenic expression of Vvi-miR3633a confirmed this result. The *Arabidopsis* transgenic strain had no difference from WT in normal growth and development, but the survival rate of *Arabidopsis* transgenic strain under thermal treatment was lower than that of WT (**Figure 7**).

## Conclusion

In summary, we assessed the heat resistance of 38 germplasm resources. Subsequently, the leaves of the heat-resistant variety “Shenyue” were utilized as experimental materials for miRNA transcriptome analysis using high-throughput sequencing technology, and a series of differentially expressed miRNAs were found. This study represents the first analysis of miRNAome and potential miRNA molecules in the leaves of heat-resistant varieties. The impact of heat stress on grapevine growth and development was deliberated upon providing a theoretical foundation for understanding high-temperature response mechanisms. To better and fully understand the tolerance of germplasm resources to heat stress and the regulatory role of miRNA in different varieties, further studies are needed, and the sequence differences and targets genes have been found.

## Data Availability

The datasets presented in this study can be found in online repositories. The names of the repository/repositories and accession number(s) can be found in the article/**Supplementary Material**.

## Supplementary material

Supplementary Figure S1 Normal growth state of 38 grape germplasm resources.

Supplementary Figure S2 The heat resistance distribution plot of grape germplasm was constructed by Fv/Fm **(A)**. Cluster number error function of Fv/Fm **(B)**.

Supplementary Figure S3
**(A)** The Venn graph constructed by the sequence total intersection is the number of common sequences of two groups of samples. **(B)** The length distribution of small RNAs in CK and HT. **(C, D)** Sample correlation analysis and principal component analysis of CK (CK-1, CK-2, and CK-3) and HT (HT-1, HT-2, and HT-3).

Supplementary Figure S4 Distribution of small RNAs in each sample by annotation type (total and unique).

Supplementary Figure S5
**(A, B)** The first nucleotide bias and each position nucleotide bias of miRNAs were analyzed in all samples.

Supplementary Figure S6 Schematic diagram of novel-miRNA secondary structure. *The marker represents the mature miRNA location.

Supplementary Figure S7 Fold-change of miRNAs with different expression. The red bars indicate miRNAs that are upregulated, while the green bars indicate miRNAs that are downregulated. The vertical dotted line is |FC| = 1.

Supplementary Figure S8 Survival rate of *Arabidopsis thaliana* transgenic (OE-1, OE-2) and WT under high temperature stress.
