# Supplementary material for: Assessment of heat tolerance and identification of miRNAs during high-temperature response in grapevine
Source: Front Plant Sci. 2024 Oct 22;15:1484892. doi: 10.3389/fpls.2024.1484892 (PMC11534869; doi:10.3389/fpls.2024.1484892)
Supplement: Supplementary file 1 [file DataSheet1.zip › Supplemental files/Supplemental Table S1.docx]

**Supplementary Table S1.** Sequence of primers used for the manuscripts.

| **miRNAs Stem-loop Primer sequence** | | | | | | | |
| --- | --- | --- | --- | --- | --- | --- | --- |
| miR396 | | | gtcacatcgtatcgtgaagctgcgcagctgatgtgacTAGTTCAA | | | | |
| miR3633a | | | gtcacatcgtatcgtgaagctgcgcagctgatgtgCTCTCCTA | | | | |
| miR167 | | | gtcacatcgtatcgtgaagctgcgcagctgatgtgacTAGATCAT | | | | |
| miR166 | | | gtcacatcgtatcgtgaagctgcgcagctgatgtgacGGGGAATG | | | | |
| miR-N-1784 | | | gtcacatcgtatcgtgaagctgcgcagctgatgtgGGTCCCCC | | | | |
| miR858 | | | gtcacatcgtatcgtgaagctgcgcagctgatgtgCAAGGTCG | | | | |
| miR408 | | | gtcacatcgtatcgtgaagctgcgcagctgatgtgGCCAGGGA | | | | |
| miR156 | | | gtcacatcgtatcgtgaagctgcgcagctgatgtgacGTGCTCCC | | | | |
|  |  | | | |  | | |
| **miRNAs** | | **Primer** | | | | **Primer sequence** | |
| miR396 | | Forward | | | | tgcactagcgtgGGAATGGATG | |
| miR3633a | | Forward | | | | TGCACTAGCGTGGGAATGGATG | |
| miR167 | | Forward | | | | TGCACTAGCGTGTGAAGCTGCC | |
| miR166 | | Forward | | | | TGCACTAGCGTGTCGGACCAGG | |
| miR-N-1784 | | Forward | | | | TGCACTAGCGTTGTTGGAAGTC | |
| miR858 | | Forward | | | | TGCACTAGCGTGTTCGTTGTCT | |
| miR408 | | Forward | | | | TGCACTAGCGTGATGCACTGCC | |
| miR156 | | Forward | | | | TGCACTAGCGTGTGACAGAAGA | |
|  | Reverse | | | | | GTCACATCGTATCGTGAAGCTG | |
|  |  | | | |  | | |
| Vvi-U6 | Forward | | | | CCGATAAAATTGGAACGATACAGAG | | |
|  | Reverse | | | | TCGATTTGTGCGTGTCATCCT | | |
| At-U6 | Forward | | | | CCAGAAGGATGCATATGTTGGTGA | | |
|  | Reverse | | | | GAGGAGCCTCGGTAAGAAGA | | |
| Vv-actin | Forward | | | | CTTGCATCCCTCAGCACCTT | | |
|  | Reverse | | | | TCCTGTGGACAATGGATGGA | | |
| PHB-VvimiR3633a | | | | Forward | | | CTCTCTCTCAAGCTTGGATCCCTGCTCCCATATAACGAGTGAGAGAGA |
|  |  |  |  | Reverse | | | CTAGAGGATCAATTCGAGCTCTCAACACCTAACAAAATCAAATGAAAAAA |

| **Genes** | **Forward (5’ to 3’)** | | | | **Reverse (5’ to 3’)** | |
| --- | --- | --- | --- | --- | --- | --- |
| *AT-ACA10* | | GCTGAGAGAGTGAAGAATACAGTCA | | ATAGAGATGATGCCCACAAAAAGGTGAT | | |
| *AT-ILR2* | | CGGATGATGTTGCTGCATCCTTTG | | AGGGAGGTTGCGAACTGTGCTTTT | | |
| *AT-AGC1-12* | | TTGTCCCTGCTGTCTCTTGCTTCCA | ACCAGTCCACTGCACTACCATGTCCTT | | |  |
| *VvSPL13A* | | CTTGAGGAATTTGATGAGGGAAAGA | ATAGCAGTAGTAGGATACAGATGGGGAC | | |  |
| *VvSPL9* | | TTCGGCCAACTCGTCTGAGTCG | GAACAACCCCACTACCTCTTACCTTCTTT | | |  |
| *VvSPL10* | | TGAGAACAAAGAGTTGATAAGAGTGGGC | GAAACAGATGATGCAGGATTGACAGAA | | |  |
| *VvSPL16* | | TGCTTGGGCTGGGAATATCAAAACC | AAGTTCCAGGGAGTGTGGGATTAGGG | | |  |
| *VvMYB-LIKE* | | CACATCTTCTTCACCGCCACCTTTACT | CCCACTGCTTATCGTCTTTGCCTCA | | |  |
| *Vv-SHR* | | CATTCGTTTGATTCTGCCA  GGAAGTTGA | TGGGCCATTGAGTGCATAGCGTGT | | |  |
| *Vv-ATHB-15* | | TACCCTCGACCTTGCTTCTTCTCTT | CAGATGATATGATACTGCGGACATACTG | | |  |
| *VvHOX32* | | CAGCACCAACAACAGCAAAACCCA | CGATTCCAATAGAATCCGGACCAGG | | |  |
| *VvLRR-LIKE* | | TGGATTTGTCCACCCCAAGG | GGAGAGTGCGGTTCTTCTCA | | |  |
| *VvLRR* | | GCTTTCATAAATCTCGTAATACTCAACT | TGCAAGGTCTAAGACATGTAGGGAG | | |  |
| *VvPIF4* | | AGCAATCAAGTTGTGAATGAGGCTGACT | TTCTCCCAAAACTACTGCCCGACC | | |  |
| *VvRAD1* | | TACCCTCGACCTTGCTTCTTCTCTT | CAGATGATATGATACTGCGGACATACTG | | |  |
| *VvSF9* | | CGTTGGAGGCTCTGCTGGCTG | CTGTTCCTTACCGCTTTGATACACTTTG | | |  |
| *VvGRF1* | | TTTTCGTCATCCTCATCCTCACCCA | GTCACTACTCACCCCCAAACCCATTT | | |  |
| *VvGRF4* | | GCGGAGCAGAGGAGGTGGTCAG | GGGATTTGAAGGAGGTGTGAGAAGGA | | |  |
| *VvGRF8* | | TGACTGCTTCCCACTCTAATCGTTCTCT | GCCATTTCTTCCCATCTGTTCTCCG | | |  |
| *VvPHO84* | | TTTGTTTGGCGGATTGTCCTCATGT | CTGGGAGTTACTGGAATTTTCGGTTTTG | | |  |
| *VvGA2ox3* | | AACAACACATCTGGACTGCAAATCTCT | ACCCTATGCCTCACACTTTTAAACCTC | | |  |
| *VvAtg36* | | GCAAAAAACAAGGCAAGAAGAGATGATG | GGGAAGGAGCGGACGCACGA | | |  |
| *VvGA3ox2* | | AGGCAAAGCATTTCAACAAGGCTC | CATTTCTTCATCACTCTCTTATTACCACCA | | |  |
| *VvARF8* | | GACATCACCCGGTTCAGCAGCTATC | CCAGACATTATTCACAAATGCCTCCC | | |  |
| *VvARF6-LIKE* | | GGCTCTCTTCATCGGGTT | GCATCTACTTCCTTGTTGGTT | | |  |
