# Supplementary material for: Assessment of heat tolerance and identification of miRNAs during high-temperature response in grapevine
Source: Front Plant Sci. 2024 Oct 22;15:1484892. doi: 10.3389/fpls.2024.1484892 (PMC11534869; doi:10.3389/fpls.2024.1484892)
Supplement: Supplementary file 1 [file DataSheet1.zip › Supplemental files/Supplemental Table S2-Table S5.docx]

**Supplementary Table S2.** Fv / Fm classification results based on optimal segmentation method

| Cluster number | Error function | Optimal segmentation levels |  |  |
| --- | --- | --- | --- | --- |
| 2 | 0.7453 | 1-17,18-38 |  |  |
| 3 | 0.3184 | 1-3,4-23,24-38 |  |  |
| 4 | 0.1338 | 1-3,4-13,14-27,28-38 | |  |
| 5 | 0.0897 | 1-3,4-12,13-19,20-27,28-38 | |  |
| 6 | 0.0598 | 1,2-3,4-12,13-19,20-27,28-38 | |  |
| 7 | 0.0427 | 1,2-3,4-12,13-19,20-27,28-34,35-38 | | |

**Supplementary Table S3.** Statistical table of sample sequence in two libraries

| Sample | Raw Reads |  | Raw Reads（average） | | Clean Reads | Clean Reads（average） |
| --- | --- | --- | --- | --- | --- | --- |
| CK_1 | 16552907 |  |  | 11516077 | |  |
| CK_2 | 14918243 |  | 15335614 | 10729045 | | 11020874 |
| CK_3 | 14535691 |  |  | 10817499 | |  |
| TS_1 | 16505839 |  |  | 12708080 | |  |
| TS_2 | 17557550 |  | 16209648 | 15479225 | | 13663198 |
| TS_3 | 14565556 |  |  | 12802288 | |  |

**Supplementary Table S4.** Rfam classification statistics in each of the two libraries (total)

| **Sample** | **Known miRNA** | **Novel miRNA** | **rRNA** | **snoRNA** | **snRNA** | **tRNA** | **unknown** |
| --- | --- | --- | --- | --- | --- | --- | --- |
| CK-1 | 383069 | 10151 | 6870520 | 63440 | 13085 | 352273 | 3823539 |
| CK-2 | 303064 | 8617 | 6296162 | 55950 | 13898 | 326252 | 3725102 |
| CK-3 | 324413 | 8771 | 6252354 | 58260 | 12223 | 345864 | 3815614 |
| HT-1 | 263065 | 7104 | 7498314 | 87536 | 17732 | 418340 | 4415989 |
| HT-2 | 223399 | 7053 | 9175849 | 182125 | 18543 | 755719 | 5116537 |
| HT-3 | 171933 | 5095 | 7583255 | 154851 | 15740 | 601950 | 4269464 |

**Supplementary Table S5.** Rfam classification statistics in each of the two libraries (Unique)

| **Sample** | **Known miRNA** | **Novel miRNA** | **rRNA** | **snoRNA** | **snRNA** | **tRNA** | **unknown** |
| --- | --- | --- | --- | --- | --- | --- | --- |
| CK-1 | 2677 | 246 | 349044 | 9402 | 4435 | 28002 | 625079 |
| CK-2 | 2437 | 258 | 366491 | 9149 | 4770 | 29581 | 638639 |
| CK-3 | 2506 | 274 | 352702 | 8759 | 4239 | 27819 | 626174 |
| HT-1 | 2398 | 232 | 429964 | 10928 | 5650 | 36119 | 768805 |
| HT-2 | 2126 | 282 | 567077 | 13022 | 6339 | 48039 | 979983 |
| HT-3 | 1908 | 228 | 492908 | 11342 | 5641 | 40614 | 835277 |
