# Supplementary figures and images for: Assessment of heat tolerance and identification of miRNAs during high-temperature response in grapevine
Source: Front Plant Sci. 2024 Oct 22;15:1484892. doi: 10.3389/fpls.2024.1484892 (PMC11534869; doi:10.3389/fpls.2024.1484892)

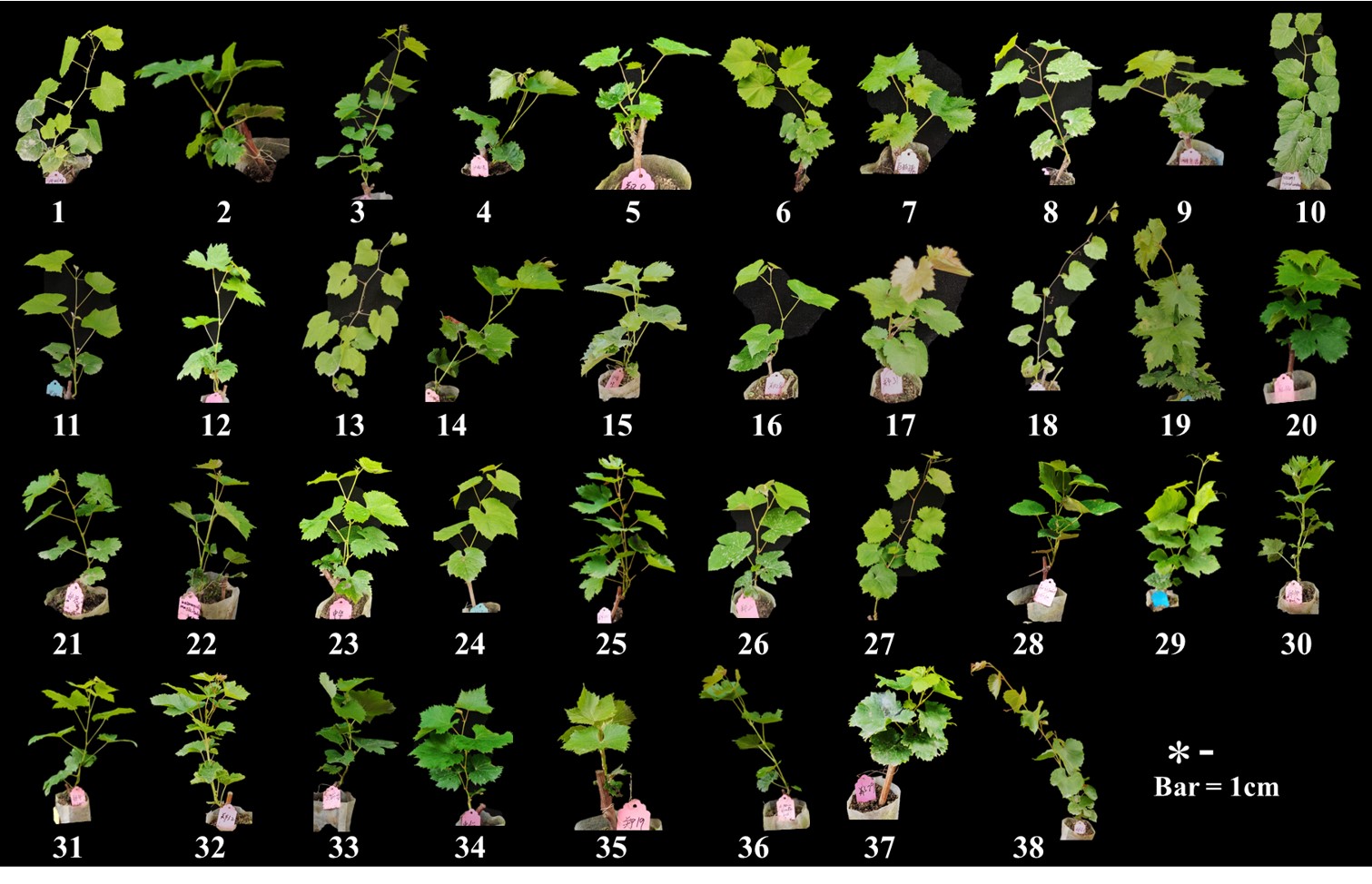

Supplement: Supplementary file 1 [file DataSheet1.zip › Supplemental files/Supplementary Figure 1.jpg]

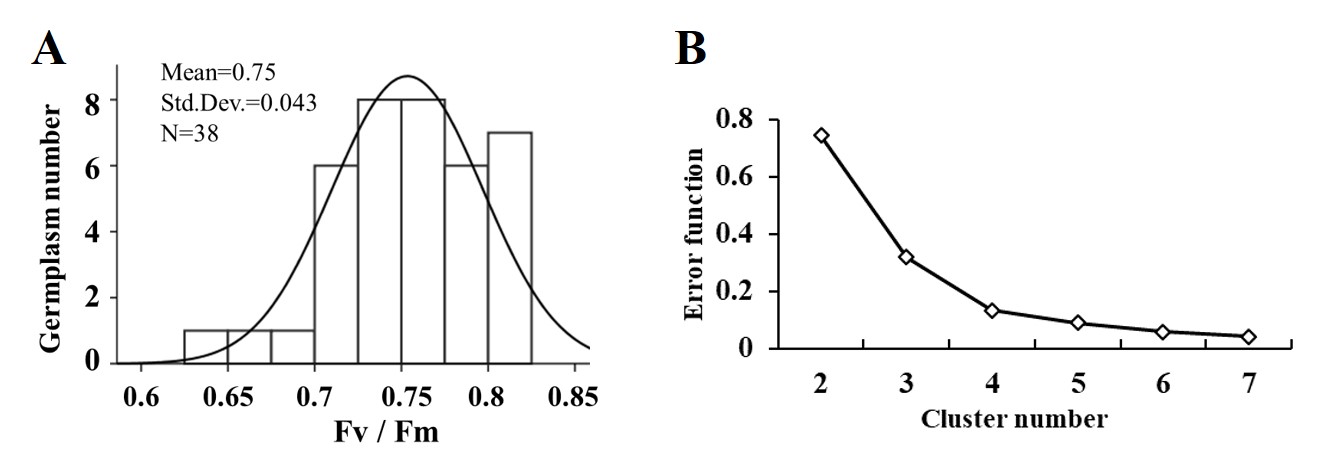

Supplement: Supplementary file 1 [file DataSheet1.zip › Supplemental files/Supplementary Figure 2.jpg]

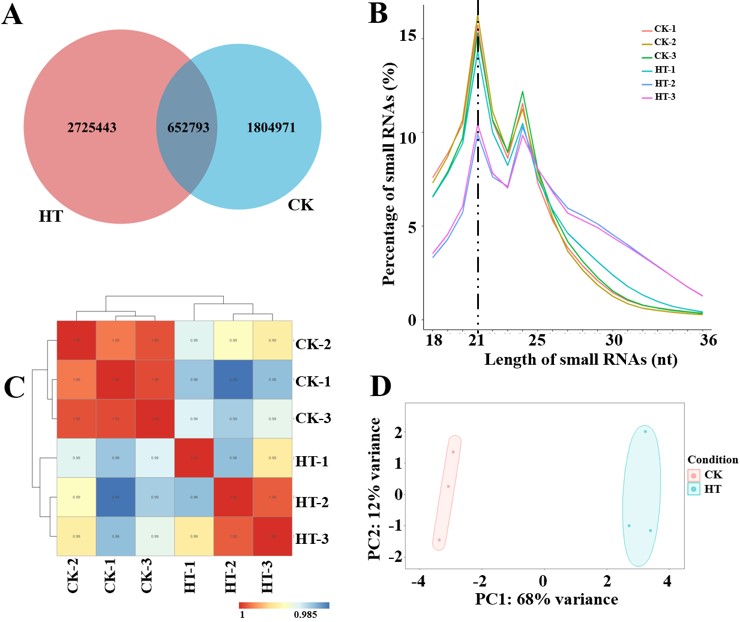

Supplement: Supplementary file 1 [file DataSheet1.zip › Supplemental files/Supplementary Figure 3.jpg]

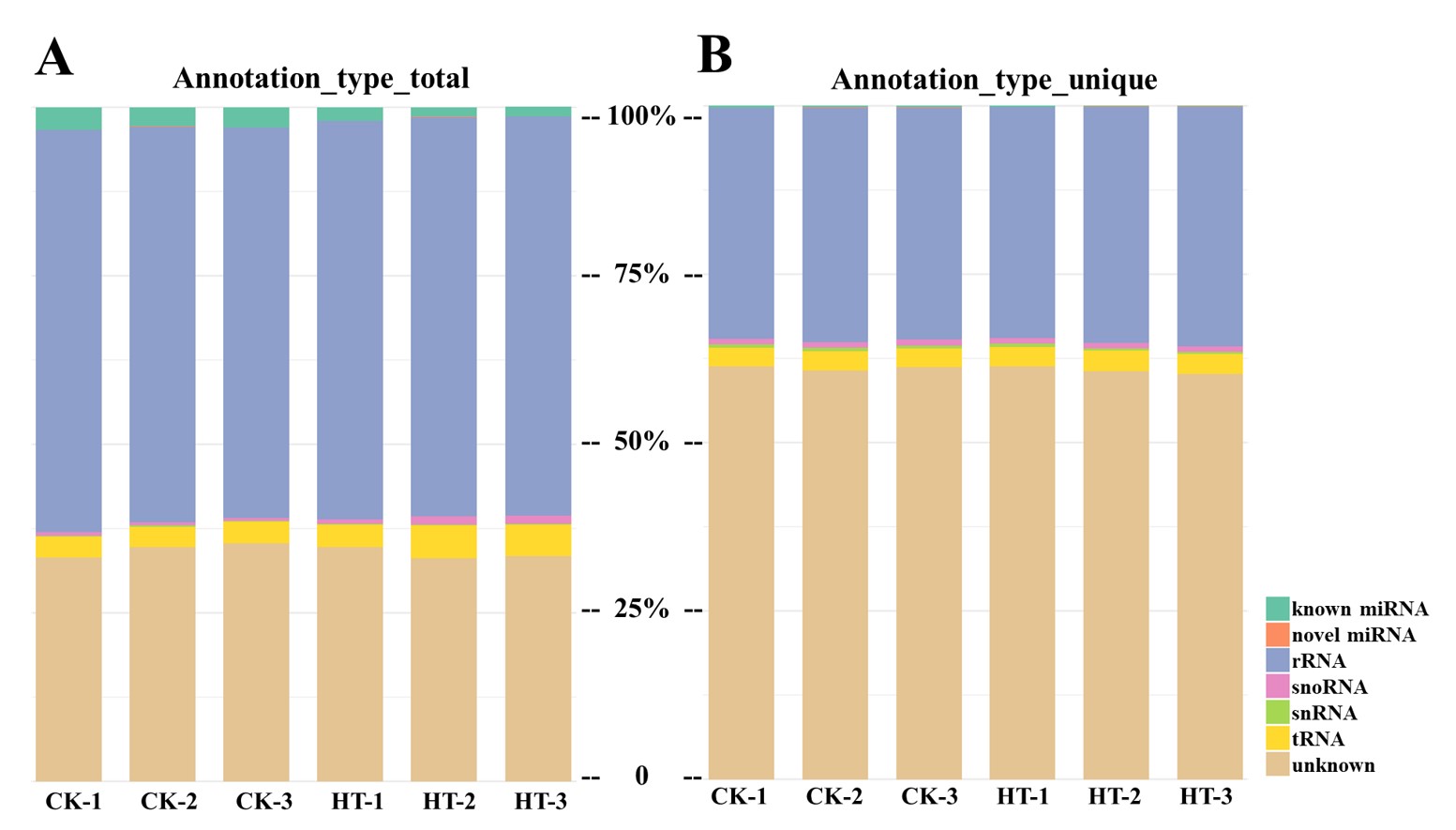

Supplement: Supplementary file 1 [file DataSheet1.zip › Supplemental files/Supplementary Figure 4.jpg]

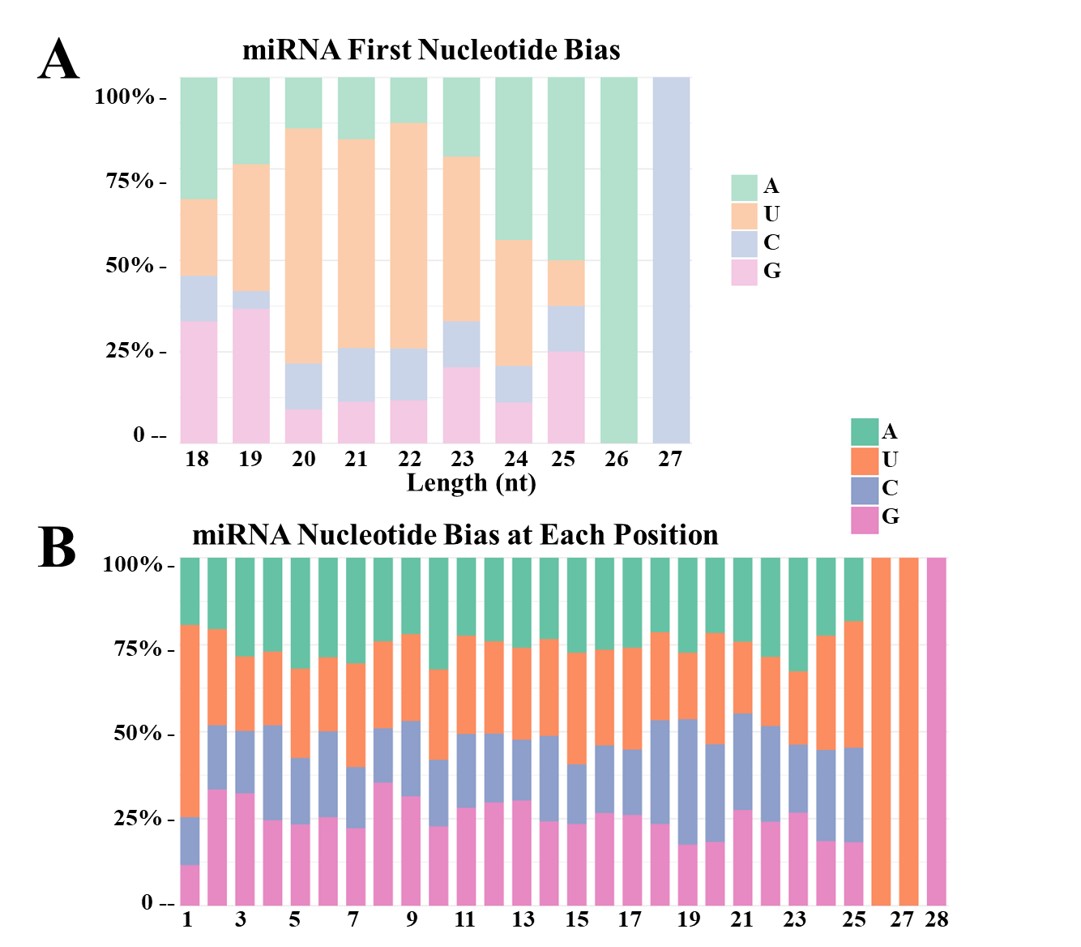

Supplement: Supplementary file 1 [file DataSheet1.zip › Supplemental files/Supplementary Figure 5.jpg]

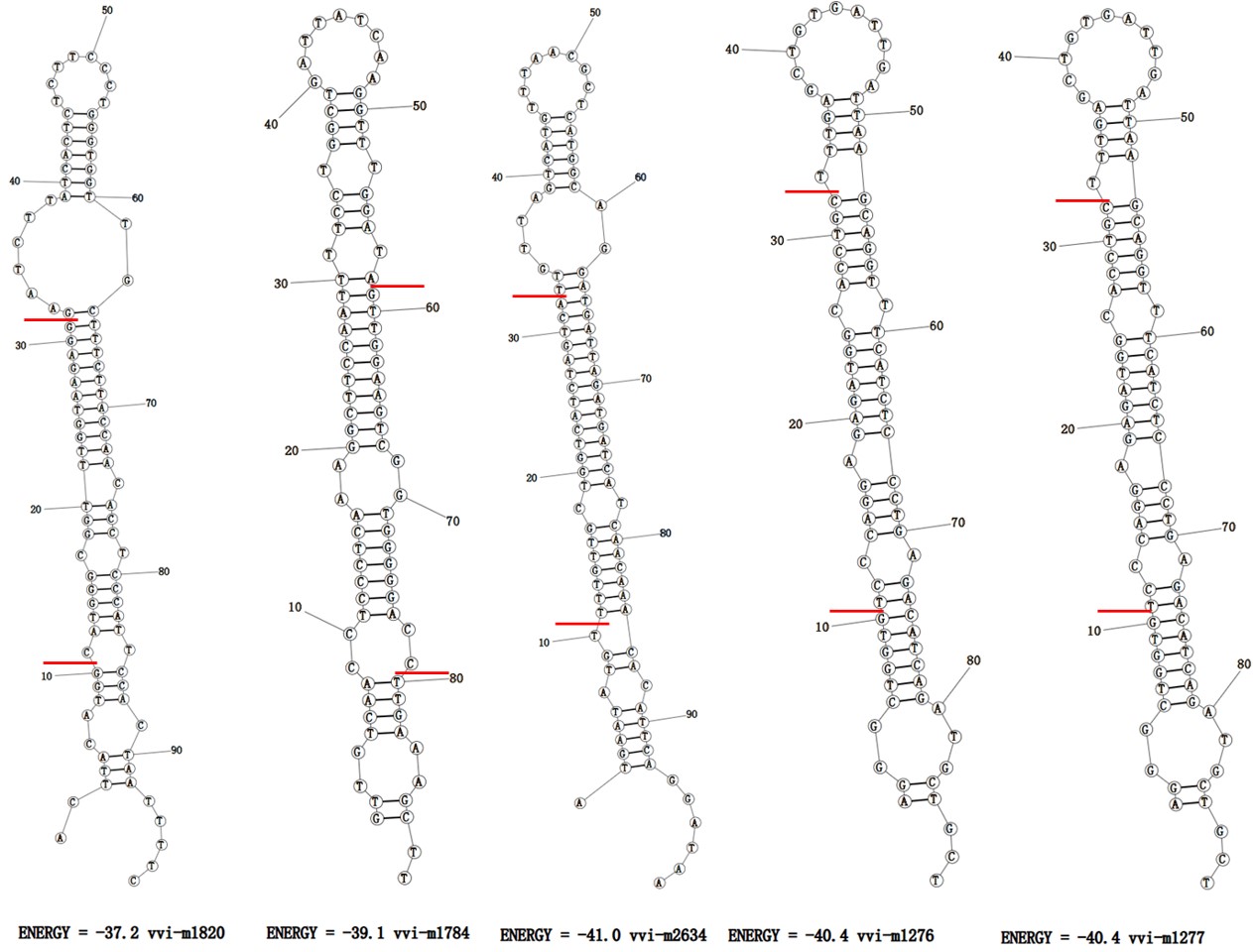

Supplement: Supplementary file 1 [file DataSheet1.zip › Supplemental files/Supplementary Figure 6.jpg]

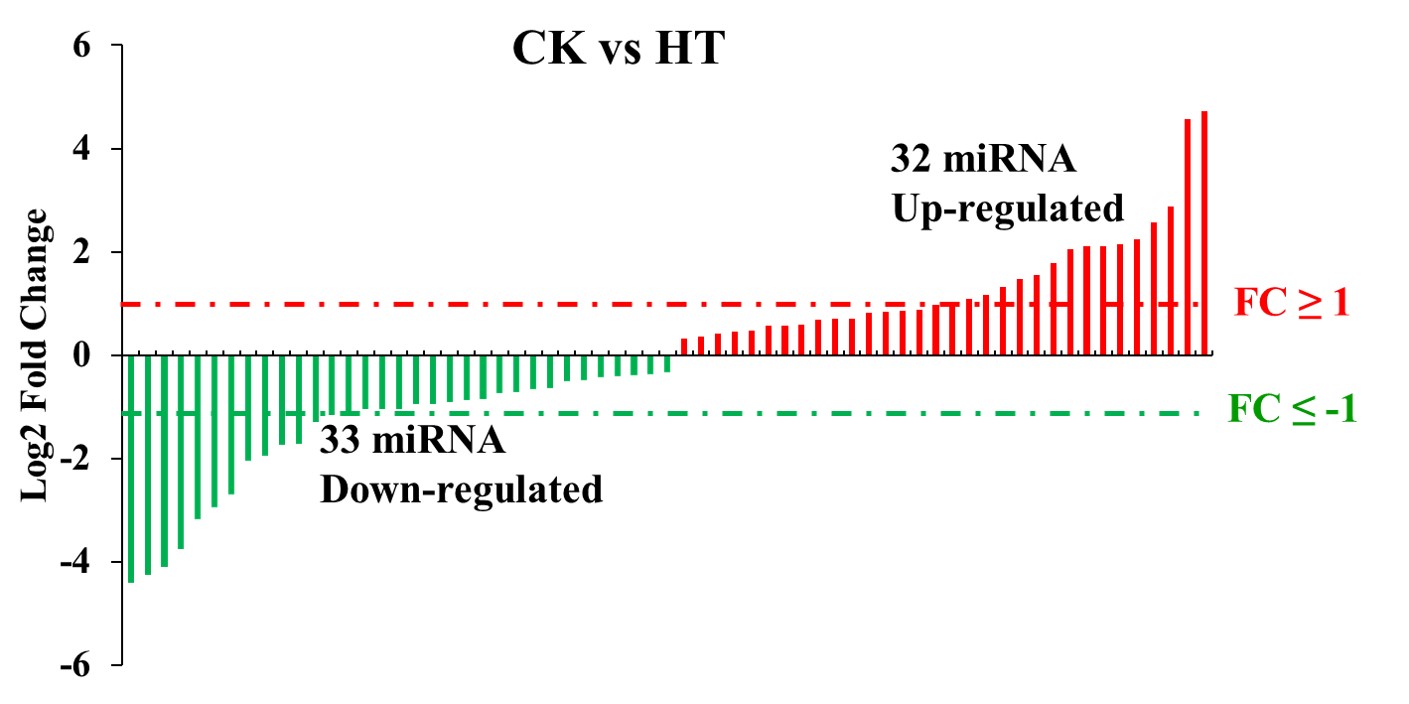

Supplement: Supplementary file 1 [file DataSheet1.zip › Supplemental files/Supplementary Figure 7.jpg]

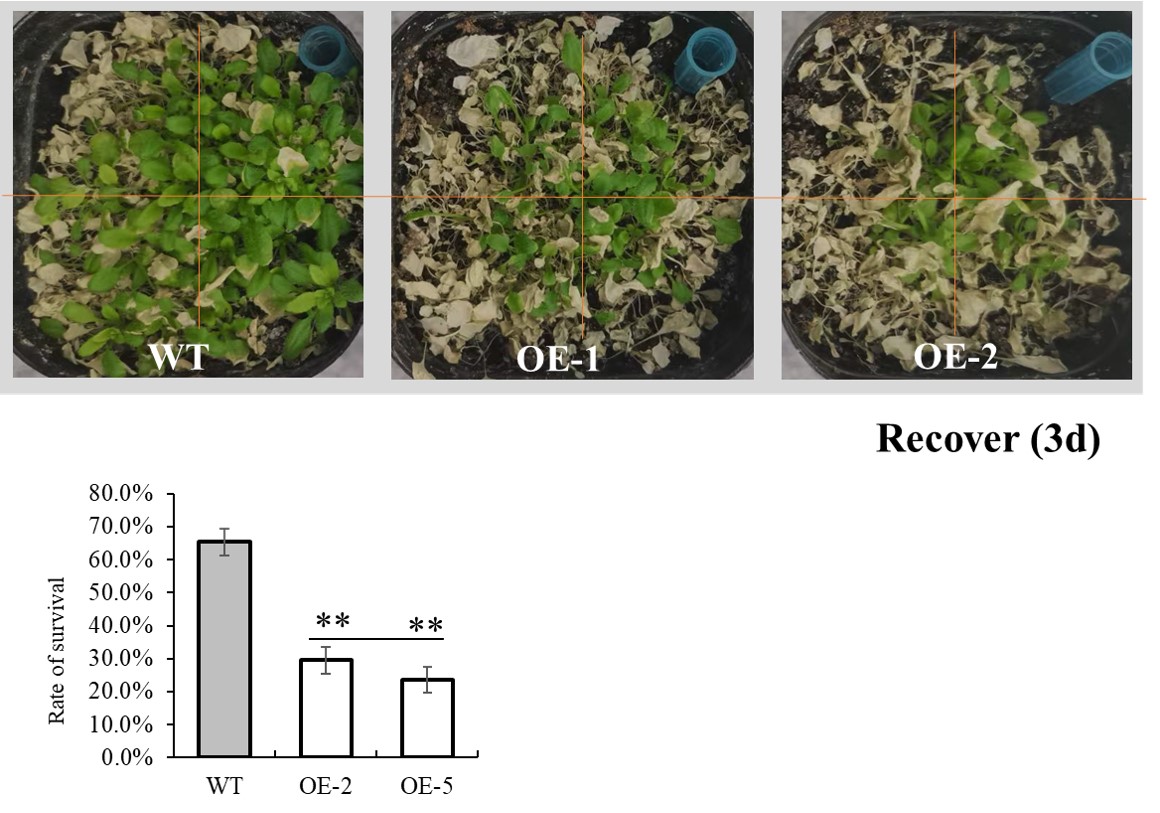

Supplement: Supplementary file 1 [file DataSheet1.zip › Supplemental files/Supplementary Figure 8.jpg]
